# Bilateral diaphyseal bone cysts of the tibia mimicking shin splints in a young professional athlete—a case report and depiction of a less-invasive surgical technique

**DOI:** 10.1186/s12891-015-0668-1

**Published:** 2015-08-23

**Authors:** Andreas Toepfer, Norbert Harrasser, Ulrich Lenze, Franz Liska, Heinrich Mühlhofer, Rüdiger von Eisenhart-Rothe, Ingo J. Banke

**Affiliations:** Klinik für Orthopädie und Sportorthopädie, Klinikum rechts der Isar der Technischen Universität München, Ismaningerstr. 22, 81675, Munich, Germany

**Keywords:** Medial tibial stress syndrome, Unicameral bone cyst, Athlete, Musculoskeltal tumor, Shin splints

## Abstract

**Background:**

Medial tibial stress syndrome is one of the most common causes of exertional leg pain in runners whereas musculoskeletal tumors and tumor-like lesions are rare encounters in orthopedic sports medicine practice.

Unicameral (simple) bone cyst is a well-known tumor-like lesions of the bone typically affecting children and adolescents. Bilateral occurrence is very rare though and has never been reported before in both tibiae. Failing to accurately diagnose a tumorous lesion can entail far-reaching consequences for both patients and physicians.

**Case presentation:**

We report the case of large bilateral unicameral bone cysts of the diaphyseal tibiae mimicking medial tibial stress syndrome in a 17-year old professional athlete. This is the first report of symmetric tibial unicameral bone cysts in the literature. The patient complained about persisting shin splint-like symptoms over 5 months despite comprehensive conservative treatment before MRI revealed extensive osteolytic bone lesions in both diaphyseal tibiae. The patient-tailored, less-invasive surgical procedure, allowing the patient to return to his competitive sports level symptom-free 3 months after surgery and to eventually qualify for this years Biathlon Junior World Championships, is outlined briefly. Pathogenesis and various treatment options for this entity will be discussed.

**Conclusion:**

This report will help to raise awareness for musculoskeletal tumors as differential diagnosis for therapy-refractory symptoms in young athletes and encourage medical staff involved in sports medicine and athlete support to perform early high quality imaging and initiate sufficient surgical treatment in similar cases. Moreover, our less-invasive surgical procedure aiming for a fast return to sports might be an optimal compromise between traditional open curettage with low risk of recurrence and a soft tissue-saving and bone-sparing minimal-invasive technique.

## Background

Medial tibial stress syndrome (MTSS), commonly known as ‘shin splints’, is one of the most common causes of exertional leg pain in runners. It is believed to be caused by a multifactorial spectrum of stress injuries including periostitis, tendinopathy, periosteal remodeling and stress reaction of the tibia [1]. Diagnosis is mostly established by patients history and physical examination. Therapy is generally conservative including rest, stretching and strengthening exercises, ultrasound therapy, iontophoresis, extracorporeal shockwave therapy (ESWT), sports compression stockings, lower leg braces and application of NSAIDs [2]. Frustrating long-term treatment intervals and delayed return to recreational and professional sports activity are feared [3]. Therapy can turn out to be protracted and thus delay individual training plans and competition. To rule out tibial stress fracture additional imaging can be obtained.

Unicameral (simple) bone cyst (UBC) is a common tumor-like lesions of the bone. There is a strong predilection for the long bones of the proximal humerus and proximal femur, accounting for up to 85 % of all cases. Symptomatic cysts are typically observed between 5 and 15 years of age with a male predominance in a ratio of 3:1 [4]. With skeletal maturation, the cyst may migrate from its initial epi-metaphyseal localization toward the diaphysis [5]. UBCs are often painless and asymptomatic and present mostly as accidental radiographic findings or pathologic fracture. Its characteristic radiological appearance with a centrally located, well-cirumscribed osteolysis and sclerotic margins allows for definite surgical therapy without the need for preceding biopsy in most cases. Relevant differential diagnoses include aneurysmal bone cyst, fibrous dysplasia and giant cell tumor and can be ruled out by MRI in most cases. Although first recognized by Virchow in 1876, etiology is still unknown. Over the decades, many theories have been proposed, but none universally accepted. Consequently, treatment still remains non-uniform. The main indication for treatment is prevention of pathologic fracture [6]. Localized at the lower extremities, UBC can cause persevering pain anf thus justify surgical therapy.

## Case presentation

**Fig. 1:**
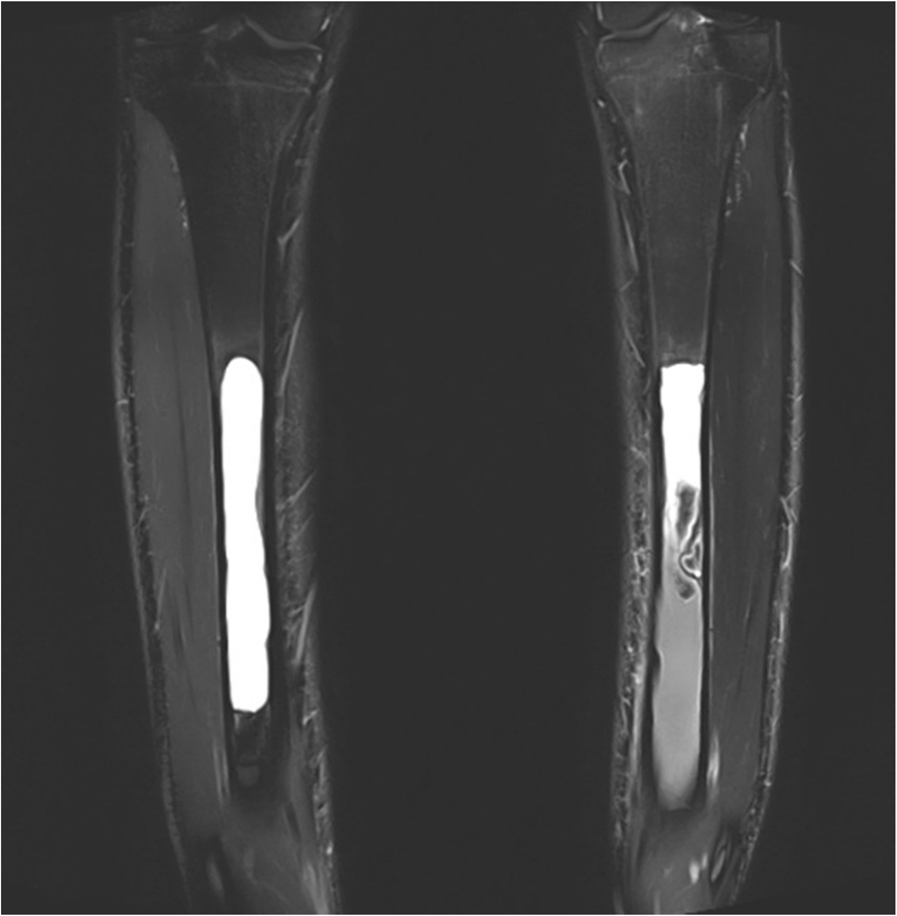
T2-weighted coronar MRI showing extensive bilateral diaphyseal cyst-like bone lesions

**Fig. 2:**
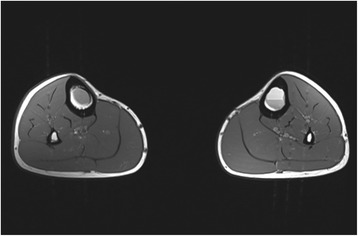
T1-weighted transversal MRI of bilateral simple bone cysts confirming its fluid content. On the left side, fluid-fluid levels created by the different densities of the cyst fluid caused by the settling of red blood cells are existent. A septation of the cyst or bloody content is more characteristic of a fractured UBC but can be interpreted as previous (repetitive) injury to the bone in this case

**Fig. 3:**
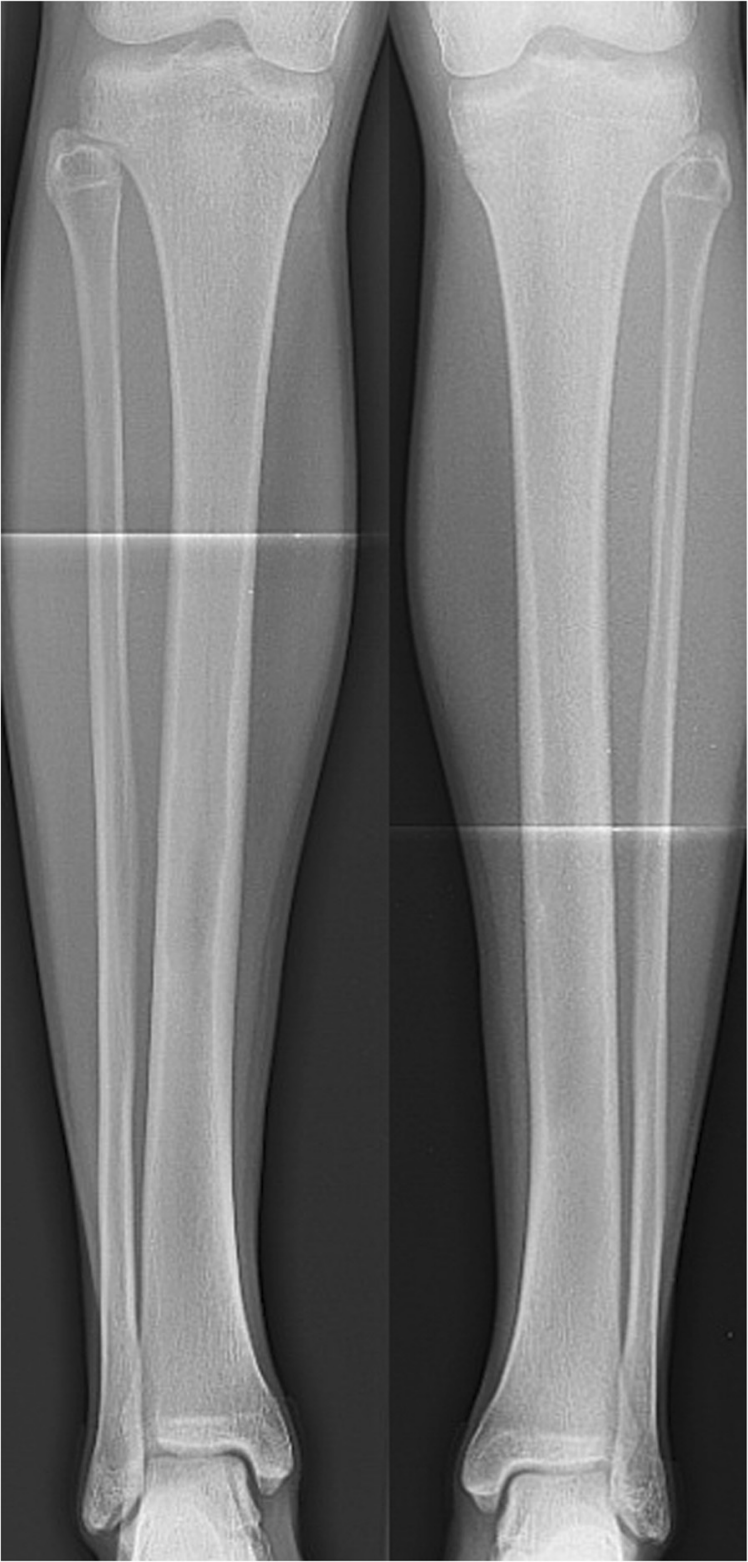
Plain radiography (a.p.) of both tibiae. Here, tender signs of reduced radiopacity with irregular cortical involvement and an expansive character of the lesion could be observed in both distal diaphyses

**Fig. 4:**
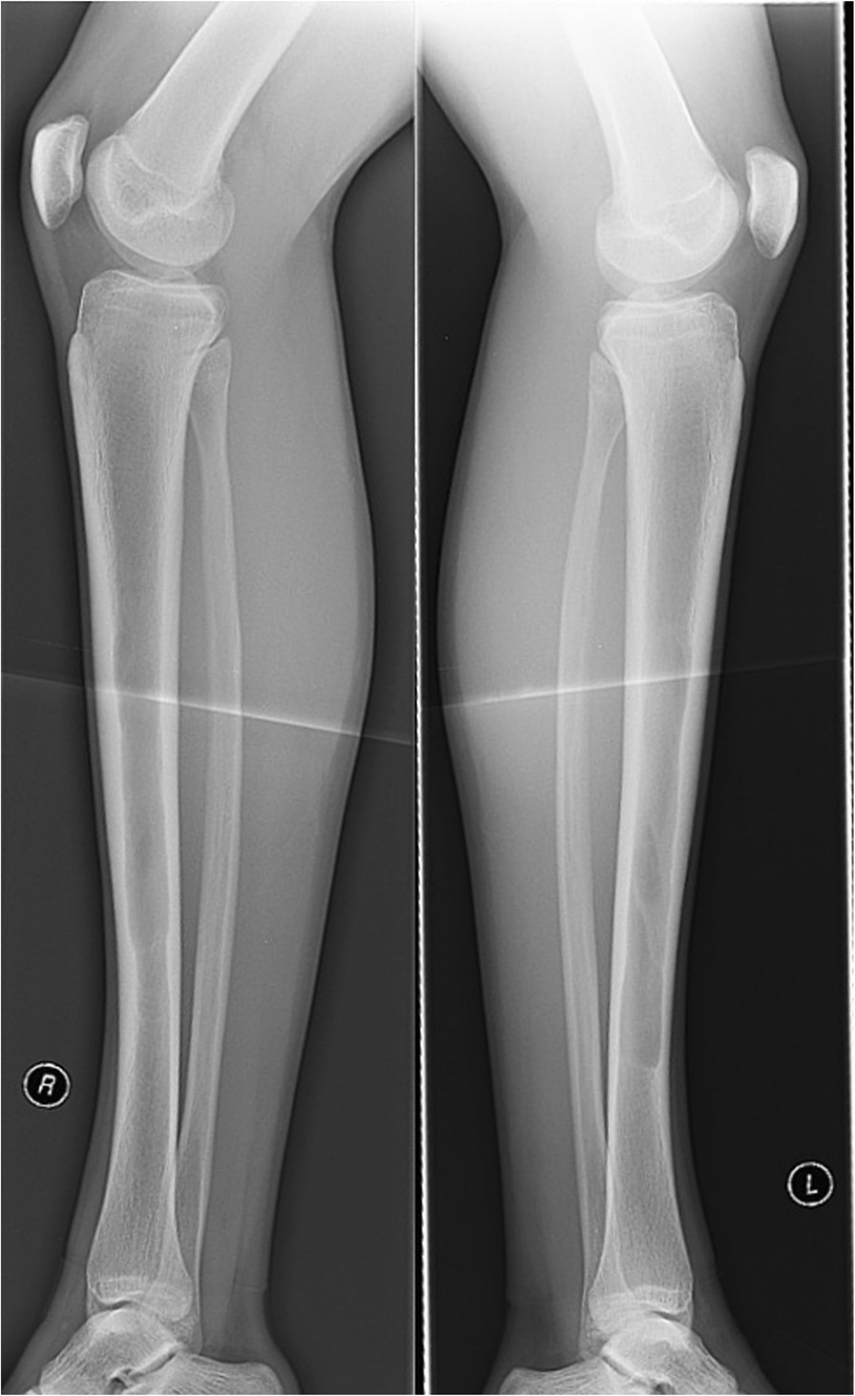
Lateral x-rays. Similar to the a.p. view, these findings would have been easy to misinterpret or miss without additional MRI

A 17-year old male professional runner was transferred to our musculoskeletal tumor centre on recommendation from his team doctor. Currently a member of a national biathlon junior team, the patient was one of the best track and field athletes of his age group for mid-distance running disciplines (1500 m & 3000 m) on a competitive level at the time of initial diagnosis. Besides daily workout in the gym for strentgh and endurance, his training schedule included up to 4 h running 6 days per week. However, ongoing pain in both of his shins prevented the young athlete from training and competition despite intensive conservative treatment for 5 months. Before admission to our hospital, symptoms were treated for shin splints without success until MRI was conducted. Extensive cyst-like bone lesiosn were found in both diaphyseal tibiae (Figs. 1 and 2). Imaging was completed by plain radiography of both tibiae (Figs. 3 and 4).

**Fig. 5:**
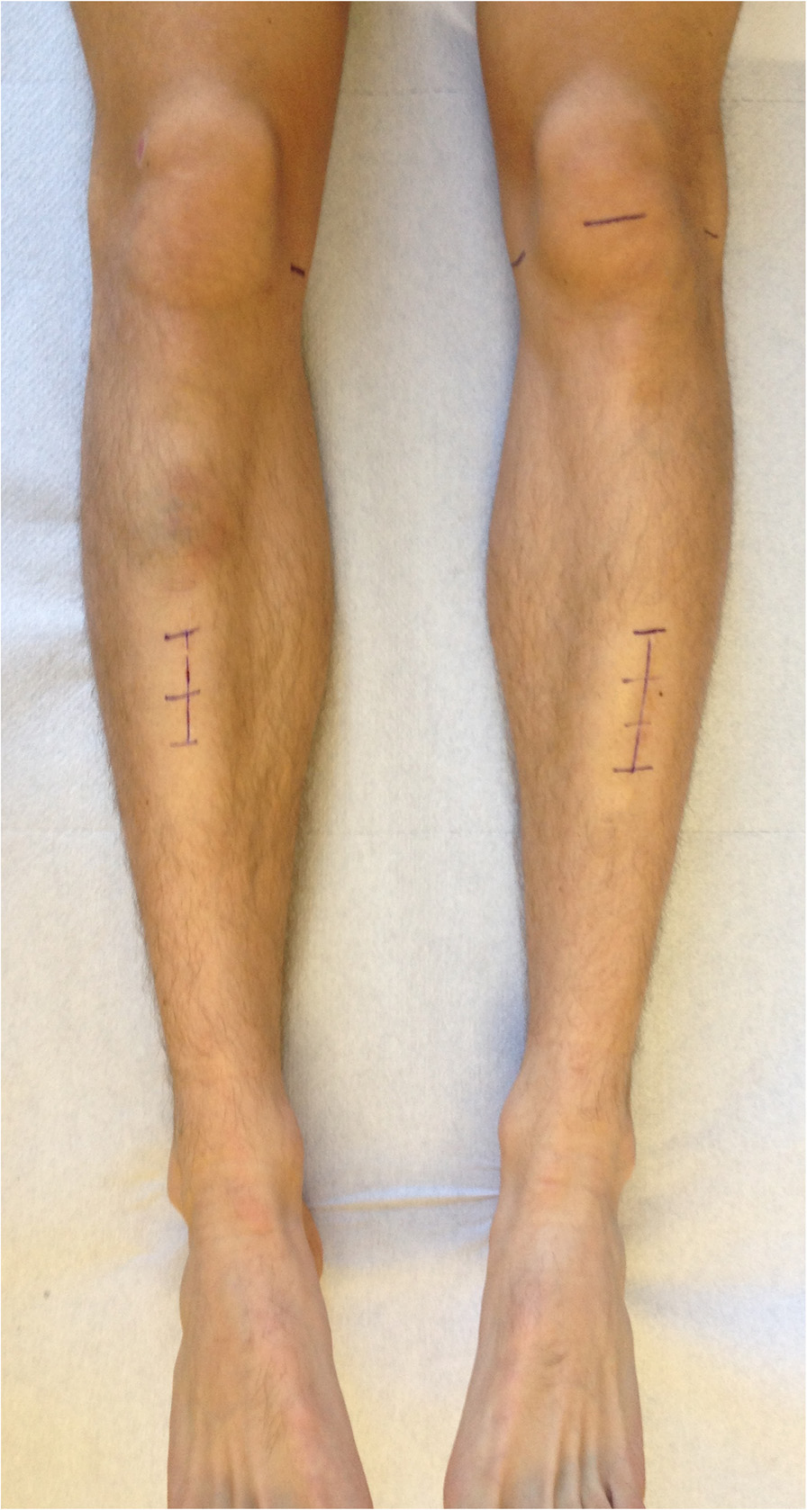
Postoperative photograph showing the length of both skin incision after less-invasive, customized intra-lesional curretage

As multimodal conservative treatment for MTSS demonstrated unsuccessful and MRI revealed the most likely reason for the patient’s symptoms, surgical therapy was indicated after thorough analysis of all clinical and radiological findings. In favour of a fast return to a competitive sports level we abandoned our standard surgical procedure for UBC consisting of open curettage and autologous cancellous bone grafting from the iliac crest. Due to the dimensions of both tibial lesions, this would have been associated with a long recovery and potential major donor site morbidity both seriously jeopardizing the young athlete’s competitive sports career. Based on promising reports of minimally invasive surgery for treatment of UBC of the humerus from Hou et al. [7, 8] we performed a customized surgical technique for the first time at the lower extremity beginning with the more symptomatic left lower leg. Surgery was conducted in a supine position without use of a pneumatic tourniquet to prevent possible muscular impairment. The exact dimension of the cyst and its distance from the lateral knee joint line and the tibiotalar joint line were marked on the skin. Intraoperative fluoroscopy was used to confirm correct localization. After a skin incision of two inches (Fig. 5) the soft-tissue layers were dissected bluntly in a longitudinal direction down to the fascia of the anterior tibial muscle. The muscle was stripped from the antero-lateral facet of the tibia with its fascia, leaving a narrow strip of fascia on the edge of the tibia allowing easy later refixation. After detachment of the periost, a small bony fenestration was created by 2 mm K-wire cortical corner drillings and connecting oscillating saw cuts. Sawcuts were placed slightly tangential to hinder the bony lid to fall through the window after curettage and grafting. Before completing all four sides of the bony window further drill holes on each side of the cranial and caudal osteotomy site were placed for additional suture cerclages for later reattaching the lid. Then the content of the cyst was aspirated and sent for cytological analysis, followed by a routinely smear test for microbiological analysis. The membrane lining and any septa within the cyst were disrupted and removed with a long flexible arthroscopic ring curette (Arthrex, Germany). Specimen of the membrane were sent for histoptahologic analysis. Fluoroscopy ensured treatment of the entire cyst. Hou et al. (Taiwan) [8] proposed the use of custom-made curved curettes and impactors. In accordance with current medical laws, we converted several surgical instruments for our needs: A 2.0 mm K-wire was fixed in a universal chuck with T-handle (Synthes, Switzerland) and its sharp end bent 60–90° to use as a scraper. Furthermore a 6.0 mm sponylodesis titan rod was bent and utilized as impactor. After repeated chemical cauterization with 95 % ethanol and intermittent irrigation with normal saline solution, grafting was performed. For grafting, autlogous bone material was not the preferred choice for obviuos reasons. Bone substitutes like calcium sulfate pellets, as suggested by several other authors, have not met our expecetations in the past, can show poor osteointegration in adult patients and can entail adverse effects [9]. Thus allogenic cancellous bone (Tutoplast©, RTI Biologics/Tutogen Medical, Neunkirchen/Germany) was administered and firmly impacted within the cyst space. Finally, the fenestration was closed by reattaching the cleaned bone cover and secured with transosseous sutures. A cannulated 6.5 mm screw was inserted caudally the small fenestration under fluoroscopic guidance to provide continuous decompression.

**Fig. 6:**
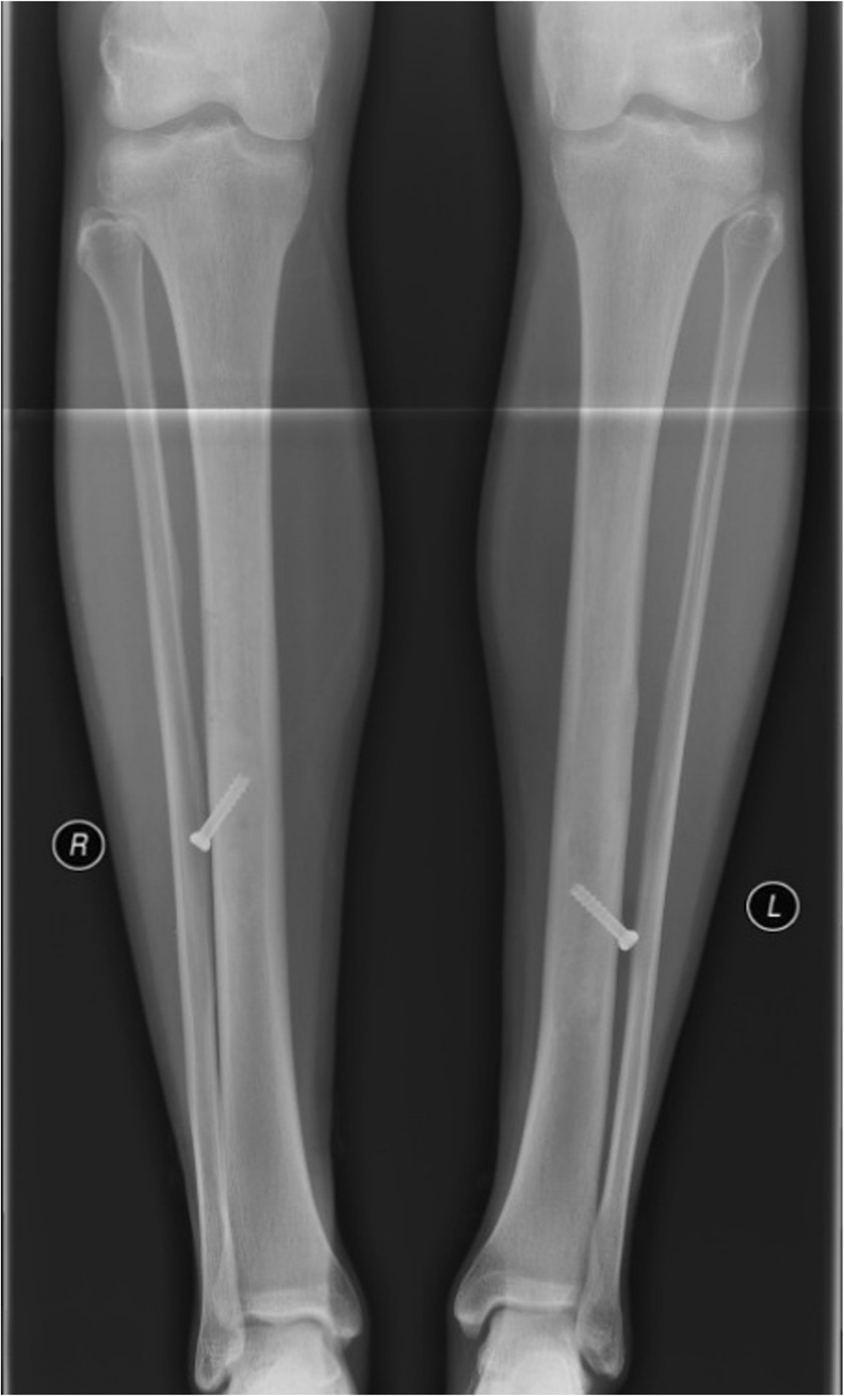
Follow-up x-rays a.p. 6 month (left leg) and 3 month (right leg) postop

**Fig. 7:**
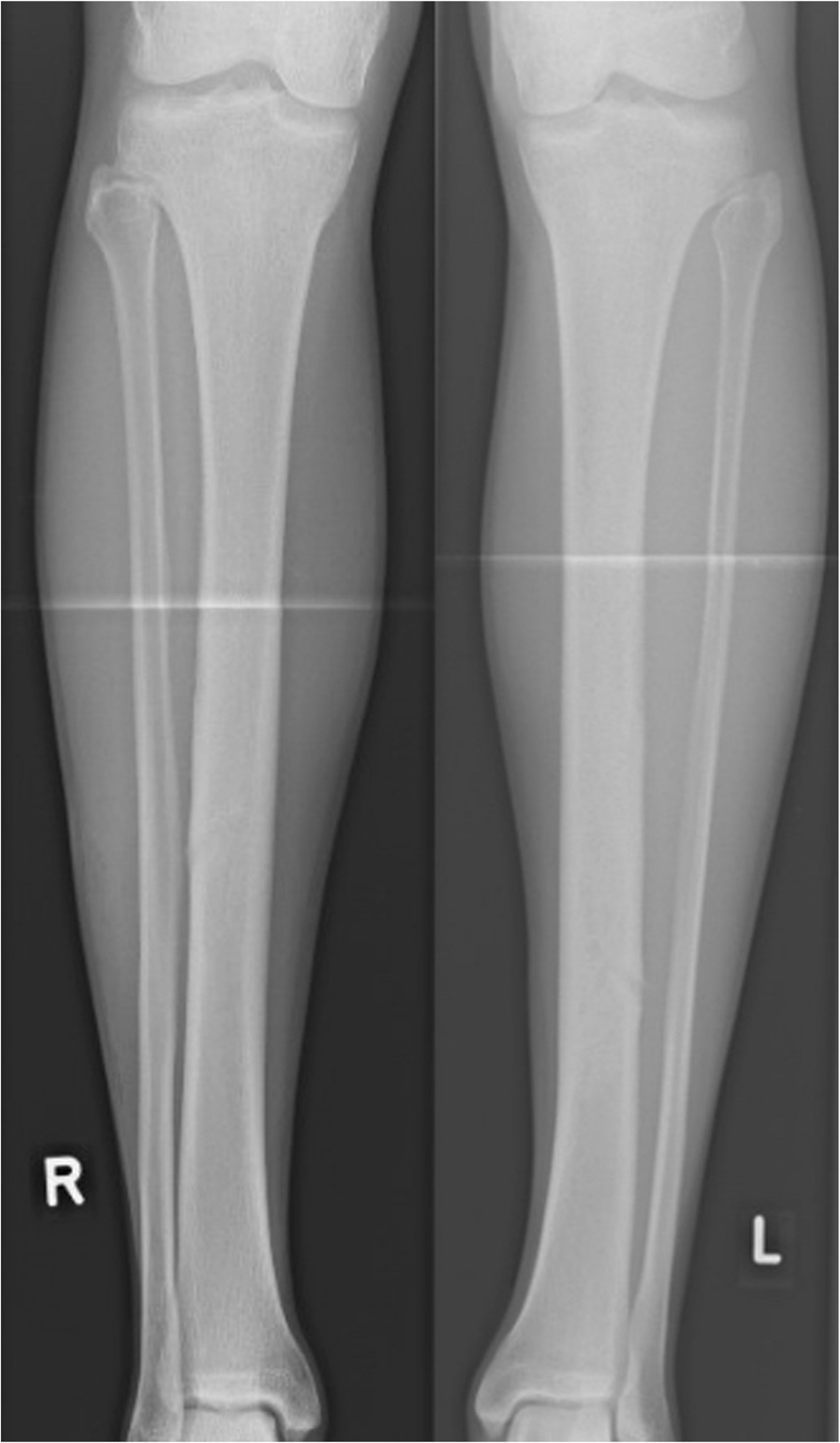
Plain radiography a.p. after percutaneous removal of the cannulated screws (34/31 months after initial surgery)

Histopathologic examination confirmed the diagnosis of UBC. Microbiology results of the smear test showed no signs of bacterial infection. After complete wound healing the patient was allowed to resume modest physical activity with cycling and swimming to regain muscular strength and decrease muscle atrophy. Six months after initial surgery on the left tibia and 3 months after last surgery on the contralateral right side the athlete had regained his full training capacity on a competive level and has been free of symptoms eversince. Clinical and radiographic follow-ups showed desirable bony consoldidation of the tibiae without signs of recurrence (Figs. 6 and 7). Three years after initial surgery, both cannulated screws were removed percutaneously. Proving the therapeutical succes of our less-inavsive procedure and showing a desirable healing process, the athlete qualified for this years Biathlon Junior World Championships in Belarus 8 month after final surgery.

## Conclusion

Etiology of UBC remains unknown. Among the accepted theories is the concept described by Cohen [10] in 1960 that UBCs have an elevated hydrostatic pressure due to developmental anomaly and therefore a decompression of the cavity is essential to obtain healing [11]. Other therories supporting a disorder of physiologic intra-osseus pressure and blood circulation include that of Chigira [12] and Watanabe [13] suggesting that increased regional blood flow, in association with bone formation, produces a hydrodynamic disorder followed by venous obstruction leading to the formation of a bone cyst. Jaffe and Lichtenstein support Mikuliez’s theory that mechanical trauma leading to a defect in ossification is the most likely cause [14]. The basic pathological process of UBC is one of bone resorption. A possible failure of prompt organization after intramedullary haemorrhage which interferes with the normal process of bony regeneration might be the initiating factor responsible for the production of that osseus cavity [15].

Regarding potential etiologic factors in the case described excessive workout during a period of rapid bone growth repetetively creating bony micro-lesions of the bone and causing a disturbance of hydrostatic pressure might have contributed to the development of UBC. Nevertheless UBC of bones of the lower extremity is not a common disorder encountered regularly in young athletes and such bilateral occurence of both tibiae is still unreported. Also other etiologic considerations have to be taken into account: Low-grade osteomyelitis as well as tumors and tumor-like lesions of the bone are suspected to act as precursor-lesion for the origination of UBC by some authors [16–18]. Bilateral appearance makes these etiologic theories very unlikely, though.

Therapeutically, radical excision of the cyst reduces the rate of recurrence but increases morbidity and complication rate [11]. Some authors even postulate open surgery to be rarely justified for the initial treatment of a unicameral bone cyst [19] proposing alternative procedures. These inlcude simple drainage of the cyst by a percutaneously inserted cannulated screw, injection of steroids into the cyst and instillation of demineralized bone matrix or bone marrow aspirate. All procedures can be combinated at the surgeons preference. Data regarding recurrence rates of the above-mentioned techniques are inconsistent. In our own experience from more than 40 years of orthopaedic oncology, a resection of the internal lining and disruption of the cystic boundary is mandatory to reduce recurrence, especially in younger patients. Our experience is shared by various other authors [6–8]. Open curretage and autologous grafting of UBCs are more and more replaced by modern techniques with a less invasive approach and thus reduced morbidity.

This is, to our best knowledge, the first report of bilateral diaphyseal simple bone cysts of the tibiae. There have been reports of two or more unicameral bone cysts in the same patient at different locations [20, 21] and one prior report of symmetric unicameral bone cysts of the hamate bones [22] as well as one report of bilateral symmetrical cysts in the proximal epiphyseal tibiae [23]. An extensive literature review from Abdel-Wanis [23] revealed 13 reported cases of multiple simple bone cysts before 2001. Nine of these 13 patients had bilateral cysts: three in the calcanei, two in femora, two in humeri, one in radius and ulna and one in carpal bones but none in the diaphyseal tibiae. Besides a case of a 13-year-old boy with bilateral distal femoral unicameral bone cysts associated with acquired generalized lipodystrophy as predisposing factor in 2010 [24], no bilateral cases of simple bone cysts outsides the field of oral and maxillofacial surgery have been published since. Notably none of the previously reported patients was a professional athlete.

Medial tibial stress syndrome (MTSS) or ‘shin splints’ is a common reason for excercise-related pain of the lower leg in young athletes, especially in running disciplines. Unless futher investigated by radiologic imaging, relevant differential diagnoses of shin splints can remain undetected. Musculoskeletal tumors and tumor-like lesions are rare encounters in sports medicine practice but have to be taken into consideration in any patient with therapy-refractory symptoms, especially in young patients. High vigilance to recognize tumor-related findings is necessary for any physician not routinely dealing with tumor orthopaedics. Additional diagnostics and definite therapy of suspicious musculoskeletal lesions should be performed in a highly experienced musculoskeletal tumor centre to prevent misdiagnosis and initiate individual treatment if required. Diagnostic errors may entail serious consequence not only for a future career in professional sports but also for life and limb of the affected patient.

### Consent

Written informed consent was obtained from the patient for publication of this Case report and any accompanying images. A copy of the written consent is available for review by the Editor of this journal.

## Abbrevations

ESWT: Extracorporeal shockwave therapy
MRI: Magnetic resonance imaging
MTSS: Medial tibial stress syndrome
NSAID: Non-steroidal anti-inflammatory Drug
UBC: Unicameral bone cyst
